# Environment and Its Influence on Health and Demographics in South Korea

**DOI:** 10.3390/ijerph13020183

**Published:** 2016-02-04

**Authors:** Ramiro D. Bravo Santisteban, Young L. Kim, Umar Farooq, Tae-Seong Kim, Sekyoung Youm, Seung-Hun Park

**Affiliations:** 1 Department of Biomedical Engineering, Kyung Hee University, Gyeonggi-do 17104, Korea; ramirobravo@gmail.com (R.D.B.S); umarf@khu.ac.kr (U.F); tskim@khu.ac.kr (T.-S.K); 2 Weldon School of Biomedical Engineering, Purdue University, West Lafayette, IN 47907, USA; youngkim@purdue.edu; 3 Department of Computer Science and Engineering, Kyung Hee University, Gyeonggi-do 17104, Korea; 4 Department of Industrial and System Engineering, Dongguk University, Seoul 04620, Korea; 5 The HIMS Inc., Gyeonggi-do 16702, Korea; parkshkhu@gmail.com

**Keywords:** self-healthcare, physical activity, U-Healthcare, neighborhood and health, environment, health promotion

## Abstract

As the prevalence of overweight and obesity has been increasing in South Korea, it is critical to better understand possible associations between environmental surroundings and general health status. We characterize key health test readings and basic demographic information from 10,816 South Koreans, obtained from two Ubiquitous Healthcare (U-Healthcare) centers that have distinct surrounding neighborhood characteristics. One is located in a rural area in Busan, the other is located in an urban area in Daegu surrounded by a highly crowded residential and commercial business area. We analyze comprehensive health data sets, including blood pressure, body mass index, pulse rate, and body fat percentage from December 2013 to December 2014 to study differences in overall health test measurements between users of rural and urban U-Healthcare centers. We conduct multiple regression analyses to evaluate differences in general health status between the two centers, adjusting for confounding factors. We report statistical evidence of differences in blood pressure at the two locations. As local residents are major users, the result indicates that the environmental surroundings of the centers can influence the demographics of the users, the type of health tests in demand, and the users’ health status. We further envision that U-Healthcare centers will provide public users with an opportunity for enhancing their current health, which could potentially be used to prevent them from developing chronic diseases, while providing surveillance healthcare data.

## 1. Introduction

Chronic diseases, such as high blood pressure and obesity, are major causes of death worldwide. Rates of obesity (body mass index ≥ 25 kg/m^2^) have increased since 2005. It is estimated that such chronic diseases affect more than 1.5 billion people over their lifetime [1]. The trend is similar in South Korea. In 2010, the Korean Center for Disease Control and Prevention (KCDC) presented the results of the Korea National Health and Nutrition Examination Survey (KNHANES), a nationwide survey aiming to understand the health and nutrition of South Koreans [2]. The KCDC reported that South Korean men were more obese than women, particularly men between the ages of 30 and 39. However, they concluded that females in their 60s had a higher rate of obesity than men in the same age category. Further, they found that rates of hypertension were higher in males than in females until the age of 50, at which age females had a higher rate of hypertension [3]. 

In 2012, another study examined the results of KNHANES and assessed the prevalence of metabolic syndrome in South Koreans. This report noted that one of five risks of metabolic syndrome was hypertension, which appeared in about 36% of South Koreans. The study found that, in general, men were at higher risk for developing metabolic syndrome than women; however, women aged 60 and above were prone to developing metabolic syndrome as well [4]. In 2013, researchers in The Korean Heart Study (KHS) investigated cardiovascular diseases across eight regions of South Korea, including Daegu and Busan, and determined that males and elderly people were more prone to cardiovascular diseases. The same study found that approximately 33% of South Korean men and 25% of women were classified as overweight or obese, and demonstrated that men in their 70s were three times more likely to develop hypertension than those in their 30s [5].

Interestingly, such a high obesity rate in South Korea may be attributable to environmental factors. One study concluded that obesity among South Koreans increased by 11% between 1998 and 2007 and state that this increase is due primarily to extensive workweeks of 60 h or more but may also be due to genetics, sleep deprivation, and the surrounding environment [6]. The impact of the environment on obesity is well known. For example, researchers in the U.S. analyzed the relationship between chronic diseases in adults (55 years or older) and these adults’ economic, social, and neighborhood environments. They found that the latter environmental factors significantly affected whether or not the adults were at risk of developing chronic diseases. Furthermore, the same study discovered that women living in economically disadvantaged areas were at greater risk of developing heart disease, high blood pressure, and diabetes [7]. In 2008, another study in the U.S. examined how neighborhoods affect obesity in elderly individuals. Neighborhood’s characteristics were important in predicting obesity by observing that those living in economically advantaged areas were less likely to be obese. In contrast, those who lived in disadvantaged neighborhoods were at a higher risk for developing obesity [8]. Also, people who lived in disadvantaged neighborhoods were more likely to develop coronary heart disease compared to those who lived in neighborhoods that had sidewalks, bicycle lanes, and green spaces, among others. Other studies documented the link between residential areas and the inhabitants’ physical activity. These studies found that inhabitants of neighborhoods with no parks or recreational facilities exercised less and were at a higher risk for heart disease compared to those living in residential areas with such facilities [9,10,11,12].

Over the past several years, to combat rising rates of obesity and other chronic diseases, healthcare professionals have shifted from making diagnoses and treating diseases to promoting healthy lifestyles and preventing maladies. To make this change, healthcare professionals utilize new services to help patients take more responsibility and monitor their health. Such a service is referred to as Ubiquitous Healthcare (U-Healthcare), “a new medical service paradigm which uses internet, mobile, among other information communication technology (ICT) in the existing medical system” to provide “medical health information, knowledge, service and products to consumers” [13].

In South Korea, the government has implemented different programs to motivate the population to increase their physical activity. The government’s investment in information communications technology has played a crucial role in providing people with access to health information that was previously unavailable to them [14]. In 2011, researchers in South Korea conducted a survey in two hospitals to evaluate awareness of U-Healthcare services among diabetic patients. More than 70% of the surveyed participants indicated their interest in using U-Healthcare services or the Internet as a means of obtaining materials that could provide them with individualized health information [15]. In another survey, researchers found that only 25.4% of surveyed participants were aware of U-Healthcare services; however, after the surveyed participants used U-Healthcare services, 96.4% found the service helpful for managing their health [16]. Another study in South Korea analyzed patients with one or more indicators of metabolic syndrome and had the patients use a U-Healthcare service. The researchers found that the number of indicators decreased during six months of using the U-Healthcare service leading the researchers to conclude that U-Healthcare services were effective in helping patients to manage health conditions [17].

In this respect, we focus on comparative empirical analyses of user’s health test status from two U-Healthcare centers located in Busan and Daegu, which have distinct surrounding neighborhood characteristics. In this study, 10,816 users visited the U-Healthcare centers and underwent several easily measurable health tests over a course of one year. First, we demonstrate that both U-Healthcare centers can provide a valuable data set to study differences in overall health test measurements between users of Busan and Daegu U-Healthcare centers. Our analyses from the data set suggest that South Koreans are interested in using U-Healthcare services to evaluate their health. Second, we investigate differences in health conditions of users, in particular blood pressure between the two centers and to compare them with relevant national health reports. Since the U-Healthcare centers were established for local residents to use on a regular basis, this cross-sectional analysis of blood pressure in the two centers may provide the idea on the influence of the environment characteristics over health outcomes. We expect this information will be highly valuable to the public and the government, because the South Korean government has allocated funds to local governments to build U-Healthcare centers which are designed to provide free health services to users, to promote wellness, and to prevent diseases.

## 2. Materials and Methods

From December 2013 to December 2014, health status data, consisting of body mass index (BMI), pulse rate (PR), body fat percentage (BF), and blood pressure (BP) were measured from 10,816 users who visited the two U-Healthcare centers. Health test evaluations were performed using HIMS Health devices. All users agreed to provide demographic information, such as age and gender, in addition to taking the health test measurements.

**Figure 1:**
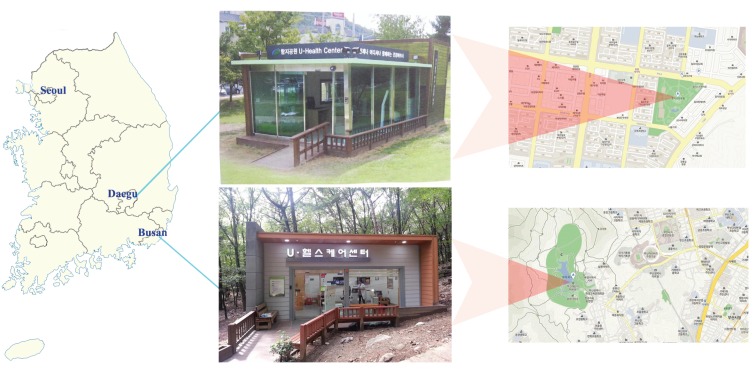
The locations of two U-Healthcare centers with photographs and their surrounding environment characteristics.

Figure 1 shows the locations of both U-Healthcare centers and their surrounding environment characteristics. The U-Healthcare center in Busan collected data from 5450 users’ health information. This center was located in Seongjigok Park (4,980,530 m^2^) in Choeup-dong, Jin-gu, Busan city [18,19]. Users needed to walk up a hill for approximately 1.2 km or about twenty minutes inside the park to access the center. The other U-Healthcare center in Daegu collected data from 5366 users’ health information. It was located in Hamji Park (46,910 m^2^) in Guam-dong, Buk-gu, Daegu city [20]. This U-Healthcare center, in contrast with the Busan U-Healthcare center, was within a few minutes walking distance from residential apartment buildings.

The U-Healthcare centers were equipped with four devices: (1) Kiosk; (2) Physical Fitness Assessment (Health Information and Management Systems [HIMS] Fitness); (3) Physical Health Assessment (HIMS Health); and (4) Mental Health Assessment (HIMS Mental). Each device processed and electronically transmitted the user’s information to the central cloud database. At each center, the kiosks were responsible for user management and synchronizing all the data with the central cloud database. Only users who chose to register with the center by using the kiosk received a radio frequency identification (RFID) card (Daegu) or barcode card (Busan) were able to access their health history through the U-Healthcare center web portal. The second and third devices, the HIMS Fitness and HIMS Health, were used to evaluate the user’s health condition. Together, these devices assessed the individual’s physical wellness. HIMS Health evaluated the most frequently used readings such as BP, PR, body weight, BMI, and BF. In addition to these readings, HIMS Fitness conducted a cardiorespiratory test and tests muscular strength, muscular endurance, and agility. The fourth device, HIMS Mental, allowed users to play digital Serious Games to evaluate the mental health of users. All these health readings were based on the American College of Sports Medicine (ACSM) [21].

To operate the devices, these two U-Healthcare centers employed Healthcare Managers (HCM) who were trained in healthcare fields such as Physical Education, Nursing, *etc.* The HCM instructed users in the appropriate use of equipment, provided education, and interpreted user’s test results. Consent from users was given verbally to the HCM.

**Figure 2:**
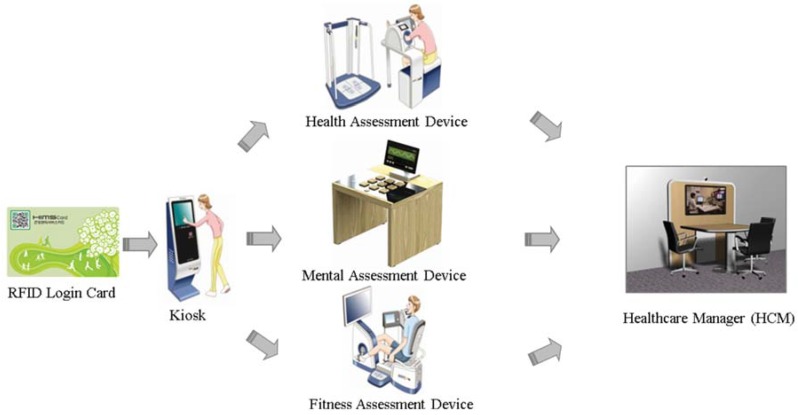
Typical flow of user accessibility to a U-Healthcare center. RFID: radio frequency identification.

When a user visited the center, the user could choose which tests to perform as shown in Figure 2. New users could register their age, gender, and password at the kiosk and received a card. If users chose not to register their information, they could still perform a test, but they must have input their age and gender at each device every time they performed a test. After the test was completed, the HCM discussed, interpreted, and educated users on how to improve their health based on their results.

Both U-Health centers were equipped to evaluate many health measurements. BP readings followed the reference categorization from the National Heart, Lung, and Blood Institute [22]. BMI readings followed the reference categorization from the American College of Sports Medicine [21]. BF readings followed the reference categorization from the American Council on Exercise [23]. PR readings were followed the reference categorization from the American Heart Association [24].

Multiple linear regression analyses were used to evaluate blood pressure as a general health indictor in the two different centers, adjusting for potential confounding factors, because the difference in blood pressure in the two centers can be distorted by other multiple variables of health tests, such as BMI, PR, BF, age, and gender. After constructing regression models, the key assumptions for linear regression, such as normality and homoscedasticity (*i.e.*, equal variances), were validated. In particular, residual analyses were conducted to validate the key assumptions. Multicollinearity was also assessed by using variance inflation factors. It should be noted that repeated measures from the same participants would be rare and unlikely to be included because the two U-Healthcare centers have recently opened. Statistical analyses were carried out using Stata (version 14, Stata Corp., College Station, TX, USA) and SPSS (version 15.0, SSPS Inc., Pompano Beach, FL, USA)).

## 3. Results

Table 1 shows the details of the gender and age demographics. Even though both centers had about the same number of users performing health tests, both had wildly different demographics. In Busan, 47.64% of users were male compared to the 70.65% in Daegu. In Busan, most users were in their 50s (40.1%) while in Daegu, most were in their 70s (51.5%). In both centers, the majority of users were older than 50 years of age.

**Table 1:** Demographic characteristics of users at U-Healthcare centers.

Factors	Age Group	Male		Female		Total	
		Count	%	Count	%	Count	%
Rural location (Busan U-Healthcare Center)							
	20~29	151	5.8%	114	4.0%	265	4.9%
	30~39	127	4.9%	232	8.1%	359	6.6%
	40~49	244	9.4%	459	16.1%	703	12.9%
	50~59	940	36.2%	1247	43.7%	2187	40.1%
	60~69	750	28.9%	574	20.1%	1324	24.3%
	70+	384	14.8%	228	8.0%	612	11.2%
	Total	2596	100.0%	2854	100.0%	5450	100.0%
Urban Location (Daegu U-Healthcare Center)	20~29	69	1.8%	72	4.6%	141	2.6%
	30~39	46	1.2%	62	3.9%	108	2.0%
	40~49	88	2.3%	228	14.5%	316	5.9%
	50~59	378	10.0%	232	14.7%	610	11.4%
	60~69	1088	28.7%	342	21.7%	1430	26.6%
	70+	2122	56.0%	639	40.6%	2761	51.5%
	Total	3791	100.0%	1575	100.0%	5366	100.0%

Table 2 presents details of the four health tests in both U-Healthcare centers. In Daegu, users were more interested in performing BP (76.01%) and PR (75.97%) tests. In Busan, most users were interested in performing BP (87.76%) and PR (87.76%) tests as well, but 40% of these users chose to perform BMI (54.42%) and BF (49.21%) tests compared to Daegu users with BMI (13.58%) and BF (9.56%) tests.

**Table 2:** Health test preferences of users at U-Healthcare centers.

Tests	Health Test in the Rural Location (Busan U-Healthcare Center)		Health Test in the Urban Location (Daegu U-Healthcare Center)	
	Count %		Count %	
Blood Pressure (BP)	4783	87.76%	4079	76.01%
Pulse Rate (PR)	4783	87.76%	4077	75.97%
Body Mass Index (BMI)	2966	54.42%	729	13.58%
Body Fat % (BF)	2683	49.21%	513	9.56%
Total	5450		5366	

Table 3 presents complete health score classifications of users in both U-Healthcare centers. The health score classification of users on BP shows that in Busan the majority of users (37.7%) had “normal BP”, while in Daegu the majority of users were classified as “right before high blood pressure” (38.9%). For PR, most users were classified as “normal” in both Busan and Daegu with 90.4% and 90.2%, respectively. For BF, the majority of users were classified as “average” with 67% in Busan and 60.2% in Daegu, respectively. The majority of users were classified as having a “normal” BMI with 63.9% in Busan and 59.4% in Daegu.

**Table 3:** Health scores at the U-Healthcare centers.

Category	Reference Value		Rural (Busan)		Urban (Daegu)	
Blood Pressure (mmHg)	National Heart, Lung, and Blood Institute (NHLBI)		Count	%	Count	%
Low Blood Pressure	Systolic < 90 or Diastolic < 60		44	0.9%	31	0.8%
Normal-Controlled	Systolic 90 to 120 and Diastolic 60 to 80		1805	37.7%	1021	25.0%
Right Before High BP	Systolic 120–139 or Diastolic 80–89		1699	35.5%	1585	38.9%
Stage 1 High BP	Systolic 140–159 or Diastolic 90–99		894	18.7%	1082	26.5%
Stage 2 High BP	Systolic ≥ 160 or Diastolic ≥ 100		278	5.8%	291	7.1%
High BP Crisis	Systolic ≥ 180 or Diastolic ≥ 110		63	1.3%	69	1.7%
Total			4783	100.0%	4079	100.0%
Pulse Rate (bpm)	American Heart Association (AHA)					
Low	<60 bpm		269	5.6%	272	6.7%
Normal	60–100 bpm		4324	90.4%	3678	90.2%
High	>100 bpm		190	4.0%	127	3.1%
Total			4783	100.0%	4077	100.0%
BMI (kg/m^2^)	American College of Sports Medicine (ACSM)					
Underweight	<18.5		288	9.7%	36	4.9%
Normal	18.5–24.9		1894	63.9%	433	59.4%
Overweight	25.0–29.9		723	24.4%	246	33.7%
Obese Class I	30.0–34.9		54	1.8%	11	1.5%
Obese Class II	35.0–39.9		7	0.2%	1	0.1%
Obese Class III	≥40		-	0.0%	2	0.3%
Total			2966	100.0%	729	100.0%
Body Fat (%)	American Council on Exercise (ACE)					
	Women	Men				
Essential Fat	10%–13%	2%–5%	4	0.1%	1	0.2%
Athletes	14%–20%	6%–13%	62	2.3%	17	3.3%
Fitness	21%–24%	14%–17%	411	15.3%	84	16.4%
Average	25%–31%	18%–24%	1796	67.0%	309	60.2%
Total			2682	100.0%	513	100.0%

**Figure 3:**
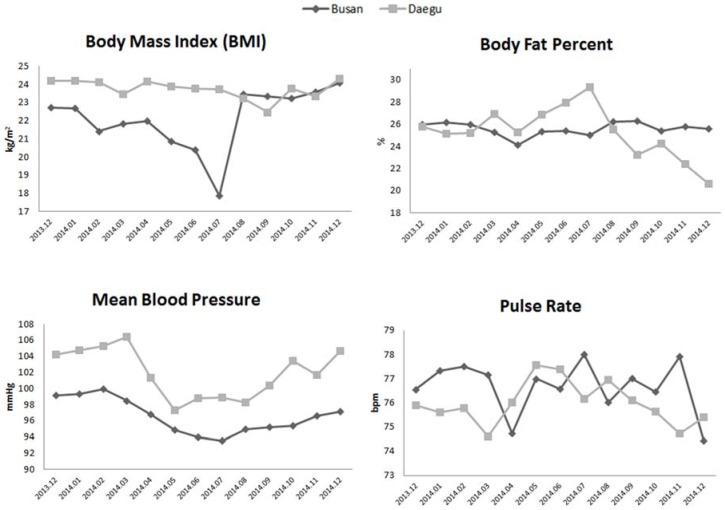
Monthly trends of health test readings.

Figure 3 shows monthly trends of the four health measurements over the four seasons at both U-Healthcare centers. Monthly trends for the four health readings were analyzed in both U-Healthcare centers in order to evaluate differences between the two centers. In Busan, the average BMI was lower than that in Daegu from January to August. BMI changes show gradual decreases starting in the spring and progressing towards the summer, while July being the lowest month compared to the average BMI in Daegu through the four seasons. BP was higher across all of the four seasons in Daegu compared to Busan.

Table 4 shows the result of multiple regression with coefficients and *p*-values. Multiple regression analyses were conducted to evaluate differences in BP between users of Busan and Daegu U-Healthcare centers, adjusting for confounding variables. The centers were coded as a binary variable (Busan center = 0 and Daegu center = 1). BP was compared in the two centers, age, gender, PR, BMI, and BFP were controlled. After adjusting for the confounding factors, BP was statistically significant difference between the two centers, which had unique environmental characteristics. Indeed, the Busan U-Healthcare center located in the rural area with the park was associated with healthier test readings than the Daegu U-Healthcare center surrounded by the highly-populated residential area with apartment buildings.

**Table 4:** Multiple regression analysis of blood pressure between two centers, controlling other variables.

Factors	Blood Pressure (BP)	
	Coef.	*p*-Value
Center	4.29	0.000
Age	0.18	0.000
PR	0.11	0.000
BMI	1.22	0.001
BF	−0.18	0.473
Gender	−1.29	0.591

## 4. Discussion

Although it would be impossible to rule out potential participant bias in the two different centers, this study provides valuable information on the association of surrounding characteristics with overall health and wellbeing. The Busan U-Healthcare center is located on a hill in a forested park. This means that users must have walked approximately 1200 meters inside the park to access the center and many of these users pass by the center on their way to a more rugged hiking trail. These types of park visitors exhibit a more active lifestyle, as evidenced by their test results. For example, the majority of the users were in their 50s and 60s, with very few users over the age of 70. Regarding BP of the Busan users, the majority was categorized as “normal-controlled” (37.7%) and “right before high BP” (35.5%), showing that few users were at risk for developing high BP. Only 26.2% of users were classified as “overweight” or “obese” and more than 60% of users had a normal BMI, meaning that the majority of users were not at risk for developing chronic diseases.

In contrast to the Busan center, the Daegu U-Healthcare center was located near residential buildings and commercial business areas. The users walked a short distance to the center on a flatter terrain. It took longer for users to reach the Busan center. Perhaps the demographics of the center’s users were influenced by those living and/or working around the center. Because of the location of this center, the users of Daegu may or may not be seeking an active lifestyle as evidenced by the user’s health test, a major contrast to the results of the Busan center. The health tests of Daegu center show that almost 78% of users were 60 years or older and were more interested in testing their BP and PR compared to Busan, where users wished to evaluate their BMI, BP, PR, and BF. In Daegu, the results show that the user’s BP was classified as “normal-controlled” (25%), “right before high BP” (38.9%), and “stage 1 high BP” (26.5%). Thus, 65.4% of users struggled with either developing or suffering from high BP. The preference of the BP test by Daegu’s older users can be attributed to the high rate of hypertension among South Koreans above the age of 70, as reported by the KHS [6]. 

While the results show that users in Daegu were older and had a tendency toward higher BP than users in Busan, the results reveal that the age of users and their environmental surroundings may play a more crucial role in determining the results of user’s health tests. In Daegu, the majority of users were males, and the data shows that these users suffered from problems with BP. In contrast, in Busan, the ratio of males to females was similar and their BP was relatively normal. This difference between the centers is not attributed to the gender discrepancy between the two centers and is more likely to attribute to the age of users, the accessibility and environmental surrounding of the center, and, perhaps, the physical activeness of users.

While other studies have proven that South Koreans are interested in using U-Healthcare services located in hospitals [16], this study indicates that they are also interested in using similar services available in recreational areas. Unlike other studies that analyzed less than 500 people [18], this study examines the data of 10,816 South Koreans who performed health tests at U-Healthcare centers. As shown in the analysis of 10,816 user’s health tests, both U-Healthcare centers would be of interest to the general population. Thus, it is expected that both U-Healthcare centers can serve as an easily accessible resource for free health check-ups supported by local South Korean governments. Among those who visited the centers, the majority of users were 50 years old and above. Interestingly, the seasons did not play a large role in determining when these users visited the facilities.

Future implementations of devices and services for targeting specific health conditions will be beneficial. In Busan, an additional service for users can include an exercise band that collects data as users walk to the top of the park’s hill. When the user returns to the center, the user’s data can be evaluated in order to provide the user with their current fitness level and help them to target their future fitness goals. For the center in Daegu, further research is needed to determine other devices and services that can be offered for users 70 years and older.

In addition, the results show that the users in Busan had less prevalence of hypertension than the users in Daegu. According to the study presented by KNHANES in 2012, the prevalence of hypertension was in about 36% of the populations. The results presented in this study show that the users in Busan had 9.6% less prevalence of hypertension than the results reported by KNHANES, while the users in Daegu had almost similar prevalence of hypertension with only 0.4% less than the results reported by KNHANES.

## 5. Conclusions

First, the study shows that South Koreans would be interested in using U-Healthcare services available in their neighborhoods. Both U-Healthcare centers in Busan and Daegu were also successful in educating South Koreans about U-Healthcare services. Using the health tests and the basic demographic information, both U-Healthcare centers provided a valuable dataset to study differences in health test measurements between users of rural and urban U-Healthcare centers, which have unique surrounding characteristics. Second, adjusting for confounding variables in multiple regression analyses, the key health variables (*i.e.*, BP) are shown to be different at the two centers. In particular, the users of the Busan U-Healthcare center in the rural area had healthier test readings than those of the Daegu U-Healthcare center, which was surrounded by apartment buildings and commercial business areas. Overall, future development of these centers will play a vital role in helping users to maintain their health and to prevent them from developing chronic diseases, while providing surveillance healthcare data.
